# Discovery of Dietary Plant Flavonols as Novel Potent Inhibitors Targeting DYRK1A Kinase

**DOI:** 10.3390/biom15070934

**Published:** 2025-06-26

**Authors:** Jin Jin, Qihong Zhou, Bin Guo, Zongchao Jia

**Affiliations:** 1 Department of Biomedical and Molecular Sciences, Queen’s University, Kingston, ON KL7 3N6, Canada; jin.jin@queensu.ca; 2 Key Laboratory of Phytochemical R&D of Hunan Province, Key Laboratory of Chemical Biology, and Traditional Chinese Medicine Research (Ministry of Education of China), Hunan Normal University, Changsha 410081, China; 202270122604@hunnu.edu.cn (Q.Z.); binguo@hunnu.edu.cn (B.G.)

**Keywords:** DYRK1A, flavonoids, protein kinase inhibitors, structure-activity relationship, molecular docking

## Abstract

DYRK1A kinase is a critical regulator in cellular signaling pathways and a promising therapeutic target for neurodegenerative diseases, diabetes and cancers. Despite its significance, the development of potent, selective and safe inhibitors remains a significant challenge. Several natural flavonoids have been reported to inhibit DYRK1A by binding in the ATP-binding pocket, exhibiting antidiabetic properties. However, a systematic screening of these structural derivatives remains lacking. In this study, we aimed to expand the pool of flavonoid-based DYRK1A inhibitor candidates for drug development against DYRK1A through targeted screening and structure-based analysis. A focused library of 13 flavonoid derivatives was screened to identify novel DYRK1A inhibitors, revealing eight new flavonol inhibitors with IC_50_ values ranging from 149.5 nM to 737.9 nM. Among these, fisetin demonstrated the highest potency with an IC_50_ of 149.5 nM, followed by kaempferol (296.3 nM), isorhamnetin (418 nM), morin (478.4 nM), myricetin (633.2 nM) and luteolin (797.8 nM), all exhibiting submicromolar inhibitory activity. Additional novel inhibitors, Apigenin and Kaempferide, also showed effective inhibition. As controls, the previously known inhibitors quercetin and curcumin were evaluated, yielding IC_50_ values of 737.9 nM and 2.35 μM, respectively, which validated the assay conditions. To the best of our knowledge, fisetin is the most potent known DYRK1A inhibitor among flavonoids. Cellular assays further demonstrated that the top flavonoid hits induced dose-dependent cytotoxicity and morphological changes in HeLa cells. Structure-activity relationship and molecular simulation analysis revealed that the selected flavonols interact with key residues for DYRK1A inhibition. These results highlight flavonols as a promising scaffold for DYRK1A inhibition and provide valuable natural inhibitor leads for further optimization and therapeutic development.

## 1. Introduction

Dual-specificity tyrosine phosphorylation-regulated kinase 1A (DYRK1A) is an evolutionarily conserved serine/threonine kinase encoded by a gene located on chromosome 21, known for its pivotal roles in regulating a wide array of cellular processes [1]. These include cell proliferation, differentiation, and neuronal development through its phosphorylation of diverse substrates such as transcription factors, splicing factors and cytoskeletal proteins [2]. The kinase’s biological significance extends beyond normal physiology, as its overexpression or dysregulation has been strongly implicated in the pathogenesis of various human disorders, such as Down syndrome, Alzheimer’s disease, neurodegenerative disorders, cancer and diabetes [3,4]. Given this broad therapeutic relevance, DYRK1A has emerged as an attractive target for pharmacological intervention across multiple disease contexts [4]. Recent studies in cancer models have revealed that aberrant DYRK1A activity can drive tumor progression and metastasis and that its inhibition significantly suppresses cancer growth in preclinical models [5]. Such findings underscore DYRK1A’s promise as a therapeutic target in diverse malignancies, as blocking this kinase not only impairs tumor cell proliferation but also enhances the efficacy of other treatments in combination therapies. However, translating this potential into effective therapies has proven challenging due to significant hurdles in inhibitor development.

Despite extensive efforts, most synthetic DYRK1A inhibitors developed to date, which have a variety of structures and show strong effects (with IC_50_ values from sub nM to µM) [6], target the ATP-binding or allosteric pocket [3], resulting in poor selectivity and significant off-target effects on other structurally related kinases, thereby compromising their safety and efficacy [7], limiting their clinical potential. Currently, increasing attention has been directed toward natural compounds as alternative sources of kinase inhibitors for chronic disease modulation. For instance, harmine, a naturally occurring β-carboline alkaloid, was one of the earliest identified potent reference inhibitors [8]. However, many of these natural DYRK1A inhibitors mainly contain nitrogen heterocycles (alkaloids) [6] and target the ATP-competitive binding site [3], thus exhibiting significant toxicity and restricting their therapeutic application [9]. Another important class of naturally occurring DYRK1A inhibitors includes polyphenols, such as catechins and flavonoids [6], which are primarily derived from dietary sources and generally meet the safety requirements of food-derived therapeutic drugs. Among them, epigallocatechin gallate (EGCG), a major polyphenol in green tea, has been identified as a potent DYRK1A inhibitor with a favorable safety profile. Although EGCG suffers from poor pharmacokinetics, various chemical modifications have been shown to enhance bioavailability [6,10,11].

Plant flavonoids, including flavonols such as quercetin, a widely distributed flavonoid compound in fruits, vegetables and herbs [12,13], represent especially promising candidates for addressing current challenges in DYRK1A-targeted therapy. These naturally occurring molecules are well known for their broad pharmacological properties, including antioxidant, anti-inflammatory, antimicrobial and anticancer effects, which have been validated by both traditional medicine and modern biomedical research [13]. Structurally, flavones feature a flat benzopyrone backbone as an ideal scaffold to interact with the enzyme active sites [8]. This structural property, combined with their biological activity, has fueled interest in their potential as kinase inhibitors [14]. Notably, several flavonoids have already demonstrated inhibitory activity against various kinases, highlighting their potential as a rich source of scaffolds for developing DYRK1A inhibitors [15].

Although several flavonoids have been reported to inhibit DYRK1A [3,8,15], a systematic screening of flavonoids for their potential to inhibit DYRK1A is still lacking. To address this gap, we aimed to systematically screen a panel of flavonoid derivatives for DYRK1A inhibition using biochemical assays and to investigate their structure-activity relationships through molecular docking and simulation analyses. We screened a library of 13 flavone derivatives, along with known inhibitors as controls, using kinase inhibition assays to explore the potential of flavones as novel DYRK1A inhibitors. Our investigation yielded promising results, with eight compounds exhibiting significant inhibitory activity against DYRK1A. The IC_50_ values of these inhibitors ranged from 149.5 nM to 3.517 μM, highlighting a broad spectrum of potencies and emphasizing the structural versatility of the flavonol scaffold for potential kinase inhibition. Further, cell viability assay and morphology analysis demonstrated that the four most potent inhibitors exhibited anticancer and pro-apoptosis effects.

These findings represent a significant step forward in the quest for effective DYRK1A inhibitors, positioning flavonols as a promising chemical class for further exploration. The identification of multiple flavonol derivatives with submicromolar potency not only enriches the repertoire of DYRK1A-targeting agents but also provides a robust foundation for subsequent medicinal chemistry efforts. The observed structural diversity among active compounds offers a valuable basis for detailed structure-activity relationship (SAR) studies, which could guide the rational optimization of potency, selectivity, and pharmacokinetic properties. Moreover, the natural origin of flavonols may offer advantages in terms of safety and tolerability, a critical attribute for advancing candidates into clinical development. By addressing the current limitations of DYRK1A inhibitors, these flavonol-derived leads hold promise as valuable tools for both basic research and drug development, contributing to the broader goal of harnessing kinase inhibition for human health.

## 2. Materials and Methods

### 2.1. Structures of Flavonoid Compounds

The natural flavonoid chemicals (with 98% purity by HPLC) tested for DYRK1A inhibition were purchased from Shanghai Yuanye Bio-Technology Co., Ltd. (Shanghai, China). All compounds were dissolved in methanol except for curcumin, quercetin, luteolin, and kaempferol, which were dissolved in DMSO. The hydroxylation patterns of each compound, based on their positions on the flavone scaffold and some molecular properties, are summarized in Table 1.

### 2.2. DYRK1A Protein Expression and Purification

The DNA encoding DYRK1A with a non-cleavable N-terminal MBP-tag was synthesized by Genscript and cloned into a pET28a vector for expression in *E. coli*. DYRK1A was expressed in *E. coli* BL21 (DE3) RIPL cells. Overnight cultures were diluted 1:500 into fresh Terrific Broth and grown at 37 °C until reaching an OD600 of 0.6. Protein expression was then induced with 0.1 mM IPTG, and the cultures were incubated overnight at 20 °C. Cells were harvested by centrifugation and resuspended in cold lysis buffer containing 20 mM HEPES pH 7.5, 300 mM NaCl, 5% glycerol, 5 mM 2-mercaptoethanol and protease inhibitor cocktail. Cell lysis was performed by sonication for 6 min using 5 s on and 15 s off cycles. Lysates were clarified via centrifugation at 18,000 rpm for 30 min. A clarified supernatant was applied to a nickel-NTA column (Cytiva 17057502). After washing with lysis buffer containing 30 mM imidazole, bound proteins were eluted with 200 mM imidazole. Fractions containing DYRK1A, as determined by SDS-PAGE, were pooled, concentrated, and further purified via size exclusion chromatography (HiLoad 16/600 Superdex 200 pg, Cytiva, Marlborough, MA, USA) in a buffer containing 20 mM HEPES pH 7.5, 150 mM NaCl, and 5 mM 2-mercaptoethanol. The purified DYRK1A was flash-frozen in liquid nitrogen and stored at −80 °C for further analysis.

### 2.3. DYRK1A Kinase Inhibition Assay

The half maximal inhibitory concentration (IC_50_) of the test compounds against DYRK1A was determined using the ADP-Glo^TM^ Kinase Assay (Promega, Madison, WI, USA) according to the manufacturer’s protocol. The peptide substrate DYRKtide (RRRFRPASPLRGPPK); synthesized by Shanghai RoyBiotech Co., Ltd., Shanghai, China, was used for the phosphorylation reaction. Briefly, 3 μL of DYRK1A enzyme (18.7 μg/mL) diluted in a buffer comprised of 50 mM MOPS, 50 mM Tris, pH7.45, 1 mM EDTA and 5 mM Sodium bisulfite was incubated with 3 μL of inhibitors dissolved in either methanol or DMSO at final concentrations ranging from 9.76 nM to 10 μM. The kinase reaction was initiated by the addition of 27 μL ATP and DYRKtide diluted in kinase reaction buffer (40 mM Tris, pH 7.5, 2 mM MgCl_2_ and 0.1 mg/mL BSA), resulting in final concentrations of 100 μM ATP and 1 mg/mL DYRKtide. The final concentration of the DYRK1A enzyme was 30 nM. The final concentration of methanol or DMSO in the reaction system was 1%. To ensure reliable measurements, DYRK1A was added immediately after dilution to minimize potential variability in activity. Given the large number of measurements and small reaction volumes, some constraints in replicates were unavoidable.

Reactions were incubated at room temperature for 1 h. At the end of the reaction, 5 μL of each reaction was transferred to a white 384-well opaque plate and mixed with 5 μL of ADP-Glo reagent. After 40 min incubation at room temperature,10 μL of the kinase detection reagent was added, followed by an additional incubation for 1 h. Luminescence was measured using a plate reader (SpectraMax iD3, Molecular Devices LLC., San Jose, CA, USA). IC_50_ values were calculated using GraphPad Prism 10. The inhibition of the test compounds was normalized to the negative group treated with only 1% methanol or DMSO.

### 2.4. Cell Lines and Culture

HeLa cells were maintained in high-glucose Dulbecco’s Modified Eagle Medium (DMEM) supplemented with 10% (*v*/*v*) fetal bovine serum (FBS) at 37 °C in an incubator with 5% CO_2_.

### 2.5. Cell Viability Assay

HeLa cells were seeded at a density of 3.3 × 10^3^ per well in 96-well plates and incubated overnight. Cells were then treated with various concentrations of flavonoids. After 48 h incubation, 10 μL of WST-8 reagent (ab228554, Abcam, Waltham, MA, USA) was added to each well and incubated for 1 h at 37 °C. Absorbance was measured at 460 nm using a microtiter plate reader (SpectraMax iD3, Molecular Devices, LLC., San Jose, CA, USA).

### 2.6. Cell Morphology Analysis

HeLa cells were seeded at a density of 1.5 × 10^5^ per well in 6-well plates and incubated overnight. Cells were then treated with different concentrations of flavonoids for 72 h. The final concentrations of methanol and DMSO were 4% and 1%, respectively. Morphology changes were observed using an Olympus IX83 (Evident Scientific, Tokyo, Japan) inverted microscope with a 40× objective.

### 2.7. Molecular Docking

Firstly, the receptor protein (PDB: 7A4O) was preprocessed using molecular visualization software PyMOL (Version 2.5.4): the water molecules and other solvent molecules and ligands were removed to eliminate their potential spatial steric hindrance or hydrogen bond on subsequent molecular docking. The active center and the size of the docking box were generated using the PyMOL plug-in GetBox (center_x = −0.8, center_y = −10.9, center_z = 12.6; size_x = 14.4, size_y 11.4, size_z = 13.9). In AutoDockTools (Version 1.5.7), the receptor protein was systematically processed to adapt to the molecular docking force field parameters: Gasteiger-Marsili charges were added first; then nonpolar hydrogen atoms of the protein were added, and lone pair electrons and non-standard amino acid side chains were removed. For the small molecule ligands, the corresponding compound’s 3D file (sdf) was downloaded from the Pubchem website (https://pubchem.ncbi.nlm.nih.gov/) and then added with charges after energy minimization (minimize) in MM2 of Chem 3D (Version 21.0.0) calculation. Finally, the receptor protein and the small molecule were exported in PDBQT format as the standardized input files for molecular docking software such as AutoDock Vina. After the docking scores were obtained, the binding modes were visualized and displayed via PyMOL, and the intermolecular interactions, such as hydrogen bonds and hydrophobic effects, were analyzed using the PLIP website (https://plip-tool.biotec.tu-dresden.de/plip-web/plip/index (accessed on 28 May 2025)).

To validate the docking results and as positive controls, we also carried out docking exercises for several known inhibitors where complex structures are available. Using PDB 6EIF as an example, the ligand was removed and docked back to the DYRK1A structure and compared to the original crystal structure. In the inhibitor-positive control, the docked position is very close to the actual X-ray structure. The calculated RMSD between the two small-molecule structures is ~1.13 Å. This low RMSD value indicates a strong structural alignment between the docked ligand and the experimental ligand.

### 2.8. Molecular Dynamics Simulation

The top inhibitor, fisetin, docked to DYRK1A, was selected for further molecular dynamics (MD) simulation analysis. MD simulations were conducted using the AMBER 19 software package. Fisetin was parameterized using the General AMBER Force Field via the Antechamber module [16,17]. To simulate realistic reaction conditions, the entire complex was solvated in a TIP3P [18] water box, with a minimum distance of 10 Å set between the protein boundary and the water box edges. Cl^−^ ions were added to neutralize the negative charges of the system. The prepared system then underwent a three-step energy minimization to gradually relieve strain from hydrogen atoms, solvent molecules, and the overall system. Following energy minimization, the system was heated to 300 K and equilibrated for 300 ps. Finally, a 300 ns MD simulation was performed under the NPT ensemble. During simulations, nonbonded interactions were truncated at 10.0 Å, and a simulation timestep of 2 fs was employed.

### 2.9. Statistical Analysis

The data analyses were performed with GraphPad Prism 10. The primary inhibitor screening data and cell viability results were presented as mean ± SEM. The inhibition curves were generated using non-linear regression, log (inhibitor) vs. response (three parameters) with GraphPad Prism 10. IC_50_ and 95% CI of IC_50_ of represented compounds were calculated using GraphPad Prism 10.

## 3. Results

### 3.1. Identification of New DYRK1A Inhibitors

**Figure 1:**
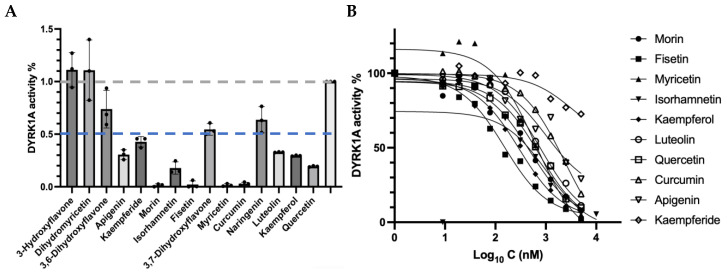
Inhibition of the selected flavone compounds targeting DYRK1A kinase. (**A**) The inhibition of selected compounds against DYRK1A kinase at 5 μM was determined in the ADP-Glo kinase assay. A 5 μM concentration of compound Quercetin was used as a positive control. Data were represented as mean ± SE of three independent experiments. 100% activity and 50% activity are highlighted with gray and blue dashed lines, respectively. (**B**) Inhibition curves of the selected compounds targeting DYRK1A kinase.

Among the tested flavone compounds, 10 of them exhibited over 50% inhibition at 5 μM (Figure 1A). The inhibitor curve (Figure 1B) and IC_50_ value of these 10 compounds (Table 2) indicated that the flavonol fisetin exhibited the most potent inhibition of DYRK1A with an IC_50_ of 149.5 nM. Followed by other flavonols like kaempferol (296.3 nM), isorhamnetin (418.0 nM), and morin (478.4 nM). Myricetin and luteolin showed moderate inhibitory effects, with IC_50_ values of 633.2 nM and 797.8 nM, respectively. In contrast, apigenin and kaempferide demonstrated weaker inhibition, with IC_50_ values in the micromolar range (1.019 μM and 3.517 μM, respectively). Quercetin and curcumin, both previously reported as DYRK1A inhibitors [8,19], showed moderate and low inhibition in this assay, with IC_50_ values of 737.9 nM and 2.35 μM. These results suggest that flavonoid-based scaffolds, particularly those of fisetin and kaempferol, may serve as promising leads for the development of DYRK1A inhibitors.

### 3.2. Flavonoids Induced Cytotoxicity and Morphology Changes on HeLa Cells

**Figure 2:**
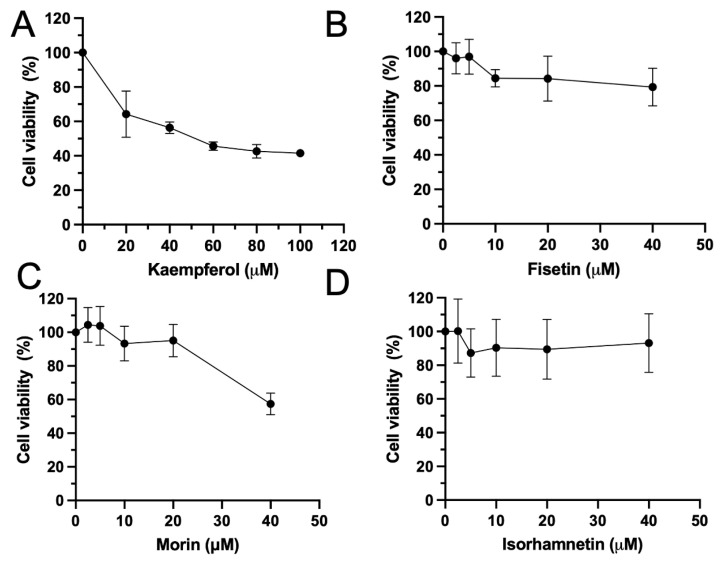
Effect of flavonoids on the cell viability of HeLa cells. HeLa cells were treated with various concentrations of kaempferol (**A**), fisetin (**B**), morin (**C**) or isorhamnetin (**D**) for 48 h. Cell viability was determined using a WST-8 reagent. The results were expressed as the percentage of cell viability compared with the vehicle control. Data were represented as mean ± SE of four independent experiments.

The results showed that after 48 h of treatment with flavonoids, kaempferol exhibited significant cytotoxicity on HeLa cells, and this effect was concentration-dependent (Figure 2A). Fisetin and morin showed a slight cytotoxicity effect on the cancer cells (Figure 2B,C). In contrast, isorhamnetin exhibited minimal cytotoxicity (Figure 2D).

**Figure 3:**
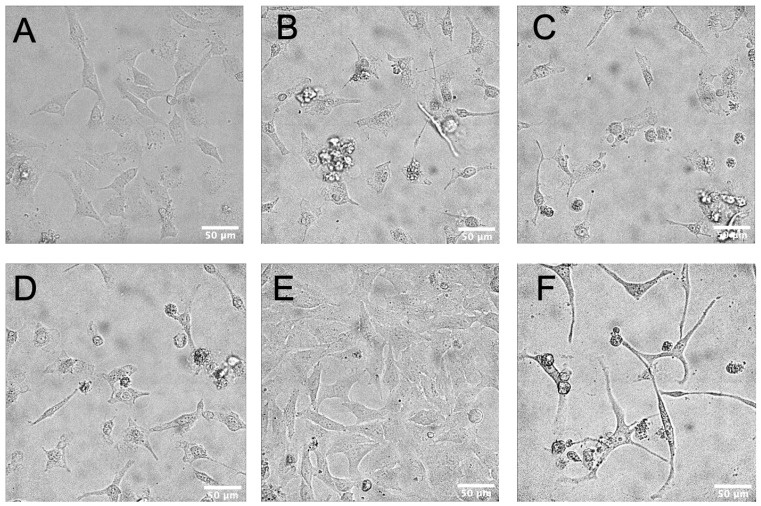
Morphological changes in HeLa cells following flavonoid treatment. Hela cells were treated with 4% methanol (vehicle control, (**A**)), 40 μM fisetin (**B**), 40 μM isorhamnetin (**C**), 40 μM morin (**D**), 1% DMSO (vehicle control, (**E**)), or 100 μM kaempferol (**F**) for 72 h. The final methanol or DMSO concentrations were 4% and 1%, respectively. Cells were visualized using an Olympus IX83 inverted microscope equipped with a 40 x objective lens. Representative images are presented. Scale bar 50 µm.

To evaluate morphology changes induced by flavonoid treatment, HeLa cells were observed under a microscope after 72 h of exposure to the four most potent DYRK1A inhibitors: fisetin, kaempferol, isorhamnetin, and morin. The negative control group, treated with 4% methanol (Figure 3A) or 1% DMSO (Figure 3E), maintained normal morphology and exhibited slight proliferation. In contrast, treatment with fisetin, isorhamnetin, or morin induced clear apoptotic features, including cell shrinkage, detachment, and the formation of apoptosis bodies (Figure 3B–D). These changes are indicative of late-stage apoptosis compared to the untreated control cells. Notably, kaempferol-treated HeLa cells exhibited a remarkable morphological alteration characterized by significant elongation and shrinkage (Figure 3F), suggesting potential involvement in cytoskeletal reorganization or a distinct mode of cell death.

### 3.3. Structure-Activity Relationship and Molecular Docking

**Figure 4:**
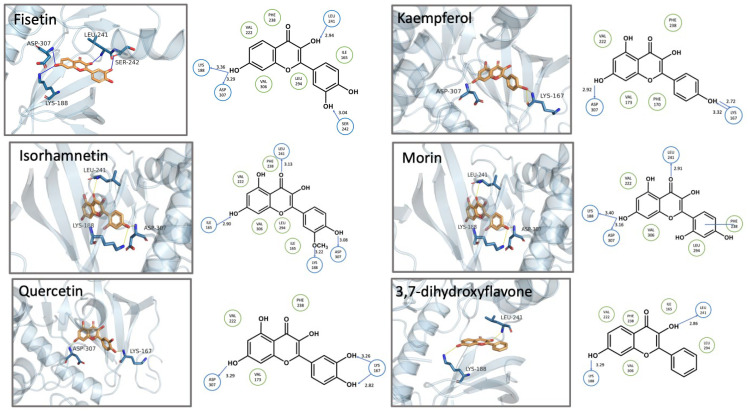
Docking poses of six flavonols bound to DYRK1A (PDB: 7A4O). The compounds (orange sticks, red sticks represent oxygen atoms) are shown bound to the ATP-binding pocket of DYRK1A (light blue cartoon representation).

The docking results indicated that fisetin exhibited the strongest binding affinity (Table 2, Figure 4) among the tested compounds, consistent with the experimental results. Fisetin formed hydrogen bonds across all three rings, featuring a well-adapted fit within the ATP-binding pocket of DYRK1A. Specifically, the 7-OH group formed dual hydrogen bonds with Lys188 and Asp307, anchoring the molecule in the binding site. The 3-OH group formed a hydrogen bond with Leu241 in the opposite direction, contributing to the stabilization of the flavonoid core. Furthermore, the 3′-OH on the B-ring underwent conformational rotation to form a hydrogen bond with Ser242. In contrast, 3,7-dihydroxyglavone and quercetin lacked hydrogen bond interaction on B- and C-ring, explaining weaker binding affinities and correspondingly lower inhibitory activity against DYRK1A (Figure 4). While kaempferol and quercetin displayed similar interaction patterns, the 4′-OH group of kaempferol formed stronger dual hydrogen bonds with Lys167 via donor-acceptor pairing, compared to quercetin’s single hydrogen bond at the equivalent position. This enhanced interaction likely contributes to kaempferol’s superior inhibitory activity against DYRK1A.

While isorhamnetin formed the same number of hydrogen bonds as fisetin (Figure 4), its binding conformation showed reduced stability due to an angular deviation between two key interactions: (1) the carbonyl group hydrogen bond with Leu241 and (2) the 7-OH hydrogen bond with Ile165, which were oriented in opposite directions. In contrast, morin’s 7-OH group formed a dual hydrogen-bonding interaction with both Lys188 and Asp307. However, compared to fisetin, the pie-pie interaction between the B ring and Phe238 was weaker than the hydrogen bonds formed between the 3′-OH group of fisetin and Ser242, exhibiting lower inhibitory activity.

**Figure 5:**
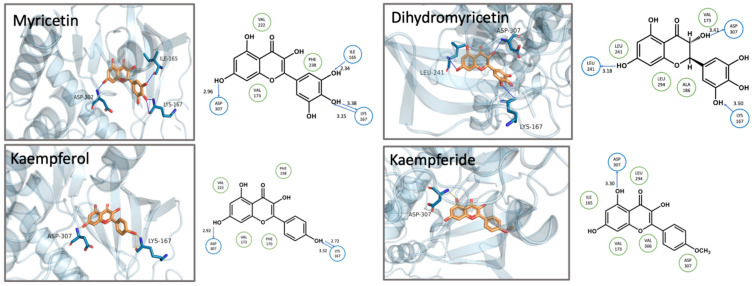
Docking poses of myricetin, dihydromyricetin, kaempferol and kaempferide bound to DYRK1A (PDB: 7A4O). The compounds (orange sticks) are shown bound to the ATP-binding pocket of DYRK1A (light blue cartoon representation).

Myricetin and dihydromyricetin share similar chemical structures but display distinct interaction patterns with DYRK1A (Figure 5). Like kaempferol, myricetin formed double hydrogen bonds through a donor-acceptor pair between 4′-OH and Lys167. The hydrogen bonds formed between 7-OH and Asp307 (2.96 Å), as well as 5′-OH and Ile165 (2.34 Å) in myricetin were both shorter than the ones formed between 7-OH and Leu241 (3.18 Å), as well as between 3′-OH and Lys167 (3.5 Å) in dihydromyricetin, indicating a stronger binding affinity and a higher inhibitory activity. Moreover, the entire molecule loses the planar structure of flavonol due to the absence of a C2-C3 double bond in dihydromyricetin.

Kaempferol and kaempferide share similar core structures; however, they exhibit significantly different interaction profiles with DYRK1A. In kaempferol, a hydrogen bond was formed between hydroxyl groups on the B ring and Lys167, while the 7-OH group interacted with Asp307, further stabilizing the molecular configuration. In contrast, kaempferide formed only a hydrogen bond between the 5-OH group and Asp307, exhibiting more conformational flexibility within the active site and less stability, contributing to lower inhibitory activity.

Myricetin and kaempferol exhibit highly similar molecular interactions, both forming hydrogen bonds with Asp307 and Lys167. In both cases, the A ring enters the active site pocket first. However, the hydrogen bond length with Lys167 is shorter in kaempferol, indicating that it penetrates deeper into the protein’s active site and occupies the binding pocket more effectively, which may explain its higher inhibitory activity compared to myricetin. In myricetin, the adjacent hydroxyl groups at the 4′ and 5′ positions on the B ring exhibit a high degree of electron density, leading to deviation of the hydrogen bond angle from the optimal range, thereby affecting binding affinity. A similar situation is observed with isorhamnetin. Although the 3′ position contains a methoxy group instead of a hydroxyl group, it still forms a hydrogen bond with Lys188, and the 4′-OH forms a hydrogen bond with Asp307. However, the presence of adjacent hydrogen bonds may also affect bond angles, potentially reducing the effectiveness of isorhamnetin compared to kaempferol, which has fewer hydrogen bonds but exhibits better activity.

### 3.4. Molecular Dynamics Simulation of DYRK1A-Fisetin Complex

**Figure 6:**
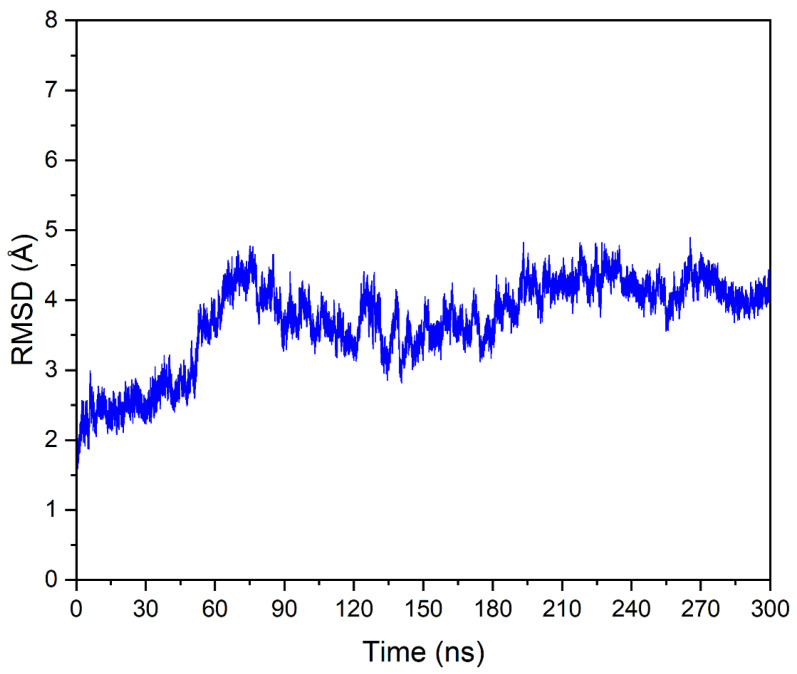
RMSD plot of the DYRK1A-fisetin complex during molecular dynamics (MD) simulation. The RMSD values are plotted against simulation time over 300 ns, illustrating the initial equilibration period and subsequent stability.

To further evaluate the stability and reliability of the DYRK1A-fisetin complex predicted by Autodock Vina docking, we performed a long MD simulation for 300 ns. The Root Mean Square Deviation (RMSD) of the complex was monitored throughout the simulation. After an initial equilibration period of ~50 ns, the RMSD values experienced no further increase and stabilized, fluctuating within a narrow range for the remainder of the simulation (Figure 6). This stable RMSD profile strongly suggests that the predicted binding mode obtained by Autodock Vina is robust, and the ligand remains stably bound in the predicted binding site without significant structural drift. The final structure obtained from the MD simulation shows an RMSD of only 1.17 Å compared to the initial docked structure, indicating that the conformation remained essentially unchanged. Thus, the combined docking and MD data suggest that the identified binding pose is energetically favorable and structurally plausible, providing a reliable basis for future experimental validation and drug development efforts.

## 4. Discussion

Flavonoids are polyphenolic compounds naturally found in a wide variety of fruits, vegetables, herbs, and medicinal plants. Their structure is characterized by a common C6-C3-C6 carbon framework consisting of two aromatic rings (A and B) connected by a three-carbon bridge that usually forms a heterocyclic ring (C). Over the past few decades, flavonoids have attracted significant scientific interest due to their significant anti-tumor activity through multiple mechanisms [20]. These compounds can inhibit cancer cell proliferation, induce apoptosis, suppress angiogenesis, and prevent metastasis across various cancer types. Additionally, flavonoids possess strong antioxidant and anti-inflammatory properties, which help mitigate the tumor-promoting effects of oxidative stress and chronic inflammation [21,22]. Their multi-targeted nature, low toxicity to normal cells, and potential to enhance the efficacy of conventional therapies make flavonoids promising agents for cancer prevention and treatment [23].

DYRK1A plays a crucial role in cellular growth, development, and differentiation and is particularly well-known for its involvement in neurodevelopment and Down syndrome [24,25]. However, in recent years, growing evidence has revealed that DYRK1A is also involved in various aspects of cancer biology, with context-dependent roles [5,26]. The anticancer mechanism includes altering cell cycle progression and increased proliferation by phosphorylating substrates such as cyclin D1 and p27 [27]. In contrast, DYRK1A can also function as a tumor suppressor by arresting the cell cycle and inhibiting proliferation. It can also enhance pro-apoptotic signaling pathways under certain stress [28,29].

In this study, we screened a library of 13 flavonoids using a DYRK1A kinase inhibition assay. Among the compounds tested, the flavonol fisetin demonstrated the highest potency (IC_50_ = 149.5 nM), followed by other flavonols like kaempferol, isorhamnetin, morin, myricetin, and quercetin. In contrast, compounds such as dihydromyricetin and 3-hydroxyflavone showed no detectable activity, while apigenin, curcumin, kaempferide, 3,6-dihydroxyflavone, 3,7-dihydroxyflavone and naringenin exhibited only weak inhibition of DYRK1A at the concentrations tested. Most of these compounds have been reported to possess anticancer activities. Interestingly, many of the anti-tumor mechanisms attributed to flavonoids such as fisetin, kaempferol, isorhamnetin, morin, myricetin, and quercetin closely overlap with the pathways regulated by DYRK1A.

We tested the impact of the four most potent DYRK1A inhibitors on HeLa cell viability. Cell viability was significantly reduced after the treatment with fisetin, morin, and kaempferol. The cytotoxicity induced by morin and kaempferol was dose-dependent at testing concentrations. Morphology analysis under a microscope revealed that all four flavonoids induced cell apoptosis after 72 h of treatment. HeLa cells in the fisetin, isorhamnetin, and morin treatment groups showed classic apoptosis features, such as cell shrinkage, detachment and apoptosis body formation. Interestingly, HeLa cells in the kaempferol treatment group exhibited an elongated morphology, indicating a distinct mechanism of cytotoxicity.

The most potent compound in this study, fisetin, can inhibit the growth of several types of cancer cells, including those from prostate, breast, colon, lung, and pancreatic cancers [30]. Fisetin inhibits cancer cell migration and invasion, reducing the potential for metastasis by modulating signaling pathways like PI3K/Akt, MAPK, and NF-kB, which are involved in metastasis [31]. Other flavonoids with strong DYRK1A inhibition, including kaempferol, isorhamnetin, morin, myricetin, and quercetin, exhibit similar anti-tumor abilities by inducing cell cycle arrest at the G2/M or G1 phase, thereby halting uncontrolled cell division [32,33,34,35,36], similar to how DYRK1A influences cell cycle regulators. These flavonoids suppress oncogenic signaling pathways, such as PI3K/Akt, MAPK, and NF-kB, which are also modulated by DYRK1A. Furthermore, the inhibition of angiogenesis and metastasis of these flavonoids aligns well with DYRK1A’s role in promoting these processes [37].

The structure-activity relationship (SAR) analysis of flavone derivatives against DYRK1A reveals several key structural features that contribute to kinase inhibition. Docking analysis revealed an interaction pattern between compounds and DYRK1A. These findings highlight the critical role of hydroxylation patterns and substitution positions on the flavone backbone in modulating DYRK1A inhibition. Systematic evaluation of structure-activity relationships, focusing on functional groups and hydrogen-bond interactions, uncovered key features modulating compound efficacy: (1) The number of hydrogen bonds; (2) Dual hydrogen bonds formed between a single functional group and one or more protein residues exhibit enhanced binding strength and stability compared to single hydrogen-bond interactions; and (3) Ortho-hydrogen bonds affect each other’s bond angles, causing the hydrogen bond angles to deviate from the optimal range, thereby affecting the binding stability. A notable trend emerges when examining hydroxylation on the B ring (positions 3′, 4′, and 5′). Potent inhibitors such as fisetin and quercetin possess hydroxyl groups at the 3′ and 4′ positions, a substitution pattern previously reported to enhance binding affinity via hydrogen bonding with ATP-binding pocket residues of DYRK1A [8]. In contrast, flavones lacking hydroxyl groups at these positions (e.g., 3-hydroxyflavone, 3,6-dihydroxyflavone and 3,7-dihydroxyflavone) or bearing methoxy substitutions (e.g., kaempferide) demonstrated low inhibition of kinase, supporting the notion that free hydroxyl groups at specific sites are essential for inhibitory activity [38]. Substitution on the A ring, particularly at positions 3 and 7, also appears to modulate activity. Kaempferol (3,5,7,4′-tetrahydroxyflavone) and isorhamnetin (a 3′-methoxy derivative of quercetin) retain moderate activity, whereas myricetin, with additional hydroxylation at the 5′ position, exhibits reduced potency. These findings suggest that excessive hydroxylation may increase the polarity of flavone molecules, potentially hindering their ability to interact effectively with enzyme-active sites [39]. Interestingly, curcumin, which differs structurally from canonical flavones yet shares conjugated aromatic systems, also exhibits modest inhibition. This may be due to partial mimicry of the planar flavone scaffold, although their reduced potency suggests a suboptimal fit within the DYRK1A active site [19]. The lack of activity from dihydroflavones (e.g., dihydromyricetin) further supports the importance of planarity and π-conjugation for interaction with the kinase domain [40].

Looking ahead, this work sets the stage for expanded investigations into the mechanisms of flavonol-mediated DYRK1A inhibition and their therapeutic potential in preclinical models of DYRK1A-associated diseases, such as Alzheimer’s disease, Down syndrome and cancer. Despite the promising findings in this study, several limitations are present. First, the biological relevance of DYRK1A inhibition by flavonoids remains to be validated in more cellular or in vivo models. Second, while many of the test flavonoids exhibit well-explored anticancer activities, more evidence is needed to confirm that DYRK1A inhibition is the underlying mechanism driving the anticancer activity of these flavonoids. Addressing these limitations in future studies will be essential to establish the translational value of flavonoid-based DYRK1A inhibitors and advance them toward therapeutic development.

## 5. Conclusions

This study systematically investigated the DYRK1A kinase inhibitory properties of a library of 13 flavonoids. Among the compounds screened, fisetin emerged as the most potent DYTK1A inhibitor, followed by kaempferol, isorhamnetin, morin, myricetin, and luteolin, all exhibiting submicromolar IC_50_ values. Notably, kaempferol not only inhibited DYRK1A but also showed significant cytotoxic and pro-apoptotic effects in HeLa cells.

These findings underscore the potential of flavonols as a promising chemical scaffold for DYRK1A-targeted therapy. The observed correlation between specific structural features, particularly hydroxylation patterns and inhibitory potency, reinforces the importance of targeted SAR analysis in guiding the development of more selective and potent inhibitors in the future. Fisetin, in particular, stands out as a lead compound for further investigation, given its strong DYRK1A inhibitory activity and favorable natural origin.

Overall, this work expands the repertoire of DYRK1A inhibitors with natural, drug-like properties and provides a foundation for future medicinal chemistry efforts aimed at improving selectivity, potency, and pharmacokinetics. These flavonoid-based inhibitors offer exciting potential for therapeutic intervention in DYRK1A-associated diseases, including cancer, neurodegenerative disorders, and diabetes.

## Figures and Tables

**Table 1:** Chemical structures and properties of selected flavones used as DYRK1A inhibitors.

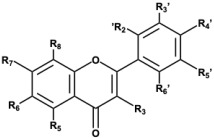 Flavones						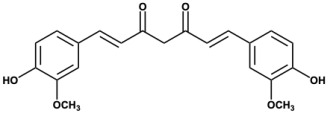 Curcumin					
Compounds	Flavone’s Substituents								Molecular Properties ^a^		
	R3	R5	R6	R7	R2′	R3′	R4′	R5′	LogP	HBA/HBD	PSA (Å^2^)
Apigenin	-H	-OH	-H	-OH	-H	-OH	-H	-H	3.04	5/3	87
Dihydromyricetin ^b^	-OH	-OH	-H	-OH	-H	-OH	-OH	-OH	1.23	8/6	148
3,6-Dihydroxyflavone	-OH	-H	-OH	-H	-H	-H	-H	-H	3.64	4/2	67
3,7-Dihydroxyflavone	-OH	-H	-H	-OH	-H	-H	-H	-H	3.27	4/2	67
Fisetin	-OH	-H	-H	-OH	-H	-OH	-OH	-H	2.52	6/4	107
3-Hydroxyflavone	-OH	-H	-H	-H	-H	-H	-H	-H	3.76	3/1	47
Isorhamnetin	-OH	-OH	-H	-OH	-H	-OCH_3_	-OH	-H	1.76	7/4	116
Kaempferide	-OH	-OH	-H	-OH	-H	-H	-OCH_3_	-H	2.74	6/3	96
Kaempferol	-OH	-OH	-H	-OH	-H	-H	-OH	-H	2.05	6/4	107
Luteolin	-H	-OH	-H	-OH	-H	-OH	-OH	-H	2.40	6/4	107
Morin	-OH	-OH	-H	-OH	-OH	-H	-OH	-H	1.61	7/5	127
Myricetin	-OH	-OH	-H	-OH	-H	-OH	-OH	-OH	2.11	8/6	148
Naringenin ^b^	-H	-OH	-H	-OH	-H	-H	-OH	-H	3.19	5/3	87
Quercetin	-OH	-OH	-H	-OH	-H	-OH	-OH	-H	2.08	7/5	127
Curcumin	-	-	-	-	-	-	-	-	2.92	6/2	93

^a^ Data from ChemSpider including ACD/LogP, hydrogen bond acceptor (HBA) number to donor (HBD) number and polar surface area (PSA). ^b^ C2 and C3 are linked by a single bond instead of a double bond.

**Table 2:** IC_50_ values and calculated affinity of the selected inhibitors.

Order	Compound	IC_50_ (nM)	95% CI		Calculated Affinity (kcal/mol)
			LogIC_50_	IC_50_	
1	Fisetin	149.5	2.012 to 2.340	102.8 to 219.0	−9.0
2	Kaempferol	296.3	2.312 to 2.633	205.1 to 429.1	−8.3
3	Isorhamnetin	418.0	2.421 to 2.823	263.9 to 664.8	−8.5
4	Morin	478.4	2.445 to 2.921	278.7 to 833.1	−8.6
5	Myricetin	633.2	2.525 to 3.097	335.2 to 1250	−8.3
6	Quercetin	737.9	2.683 to 3.058	482.2 to 1142	−8.3
7	Luteolin	797.8	2.754 to 3.055	568.2 to 1136	−8.3
8	Apigenin	1019	2.386 to 3.798	243.3 to 2681	−8.8
9	Curcumin	2351	3.238 to 3.516	1730 to 3283	−7.9
10	Kaempferide	3517	2.806 to --^a^	640.5 to --^a^	−8.4

^a^ The upper 95%*CI* value could not be determined from the available data using GraphPad Prism 10.

## Data Availability

The original data presented in this study are available on request from the corresponding author.
